# Possible Undetected Mpox Infection Among Persons Accessing Homeless Services and Staying in Encampments — San Francisco, California, October–November 2022

**DOI:** 10.15585/mmwr.mm7209a3

**Published:** 2023-03-03

**Authors:** Caroline J. Waddell, Thomas D. Filardo, Namrata Prasad, Gerald J. Pellegrini, Neela Persad, William C. Carson, Terese Navarra, Michael B. Townsend, Panayampalli S. Satheshkumar, David Lowe, Deborah Borne, Julia Janssen, Nnenna Okoye, Anamaría Bejarano, Grace E. Marx, Emily Mosites

**Affiliations:** ^1^CDC Mpox Emergency Response Team; ^2^Epidemic Intelligence Service, CDC; ^3^San Francisco Department of Public Health, San Francisco, California.

Monkeypox (mpox) is a disease caused by an *Orthopoxvirus.* The 2022 multinational outbreak, which began in May 2022, has spread primarily by close skin-to-skin contact, including through sexual contact. Persons experiencing homelessness have been disproportionately affected by severe mpox (*1*). However, mpox prevalence and transmission pathways among persons experiencing homelessness are not known, and persons experiencing homelessness have not been specifically recommended to receive mpox vaccine during the 2022 outbreak (*2*,*3*). During October 25–November 3, 2022, a CDC field team conducted an orthopoxvirus seroprevalence survey among persons accessing homeless services or staying in encampments, shelters, or permanent supportive housing in San Francisco, California that had noted at least one case of mpox or served populations at risk. During field team visits to 16 unique sites, 209 participants completed a 15-minute survey and provided a blood specimen. Among 80 participants aged <50 years who did not report smallpox or mpox vaccination or previous mpox infection, two (2.5%) had detectable antiorthopoxvirus immunoglobulin (Ig) G antibody. Among 73 participants who did not report mpox vaccination or previous mpox infection and who were tested for IgM, one (1.4%) had detectable antiorthopoxvirus IgM. Together, these results suggest that three possible undetected mpox infections occurred among a sample of persons experiencing homelessness, highlighting the need to ensure that community outreach and prevention interventions, such as vaccination, are accessible to this population.

During July–October 2022, the San Francisco Department of Public Health (SFDPH) identified cases of mpox among persons who were experiencing homelessness (Deborah Borne, MD, San Francisco Department of Public Health, personal communication, October 2022). SFDPH invited a CDC field team to conduct an orthopoxvirus seroprevalence survey among persons experiencing homelessness to better understand the prevalence of disease and possible transmission pathways. Existing collaboration between SFDPH and government- and community-based organizations serving persons experiencing homelessness facilitated site engagement in the project. Homeless service sites were prioritized for inclusion if they had provided services to at least one person with confirmed mpox or served persons at increased risk for mpox, including those identifying as lesbian, gay, bisexual, transgender, or queer; men who have sex with men; and persons who engage in sex work.

Participants provided oral consent to respond to a 15-minute orally administered survey and to provide a serum specimen. Persons aged ≥18 years were eligible to participate. The survey included questions about demographic characteristics and behaviors that could be related to mpox transmission in the context of homelessness, including frequency of sharing objects such as clothing and utensils as well as sexual activity and drug use in the preceding month. Surveys were conducted in person in English or Spanish or through a phone-based language interpreter if another language was requested. A trained phlebotomist performed venipuncture. When venipuncture was unsuccessful or when success was considered unlikely (e.g., because of needle aversion or inadequately visualized or damaged veins), participants were offered use of a microneedle device that passively collected capillary blood from the upper arm (*4*). Each participant received up to two $25 grocery store gift cards for participating in the survey and blood collection. This activity was reviewed and approved by CDC and conducted consistent with applicable federal law and CDC policy.^†^

Blood specimens were centrifuged in serum separator tubes and shipped to CDC for processing. All serum specimens were tested by enzyme-linked immunosorbent assay (ELISA) for presence of antiorthopoxvirus IgG. Those specimens with detectible IgG or from participants who reported higher mpox risk behaviors (sex work, multiple sexual partners, and persons assigned male at birth who identified as gay, bisexual, or transgender), were tested for IgM (*5*). Possible undetected mpox infection was defined as 1) detectable antiorthopoxvirus IgG (optical density minus cutoff value [OD-COV] ≥0.1) in a participant aged <50 years without reported smallpox or mpox vaccination or 2) detectable antiorthopoxvirus IgM (OD-COV ≥0.1) in a participant without reported mpox vaccination. Possible undetected infections identified by IgG testing were restricted to persons aged <50 years to avoid inclusion of participants who received smallpox vaccine during childhood. Participants who reported unknown vaccination history or previous mpox infection were not included as having possible undetected mpox infections. Descriptive analyses were performed, and outcomes were reported by vaccination history and serologic results. 

The CDC field team recruited 284 participants from 16 unique sites (seven shelters, five service centers, two supportive housing locations, and two encampments); 11 (4%) participants were excluded from analysis because they reported living in their own private residence without accessing homeless services during the preceding month. Among the remaining 273 participants, 240 (88%) consented to blood collection, and blood was successfully collected from 209 (77%) (Table 1). Average participant age was 46 years; 69% reported male sex at birth, 59% reported male gender, and 9% were transgender. Most (77%) were heterosexual. The highest proportion of participants was non-Hispanic White (38%), followed by non-Hispanic Black or African American (32%); 70% were non-Hispanic. The most common recruitment sites were shelters (46%), followed by homeless service sites (33%).

**TABLE 1:** Characteristics of persons experiencing homelessness or accessing homeless services who participated in an mpox seroprevalence survey (N = 209) — San Francisco, California, October–November 2022

Characteristic	No. (%)
**Mean age, yrs (range)**	46 (19–83)
**Sex at birth**	
Female	61 (29.2)
Male	145 (69.4)
Intersex	<5.0^†^
Unknown*	<5.0^†^
**Gender**	
Cisgender man	124 (59.3)
Cisgender woman	63 (30.1)
Nonbinary	<5.0^†^
Transgender man	0 (—)
Transgender woman^§^	19 (9.1)
Unknown*	0 (—)
**Sexual orientation**	
Bisexual	22 (10.5)
Gay or lesbian	14 (6.7)
Heterosexual	160 (76.6)
Pansexual	<5.0^†^
Unknown*	<5.0^†^
Other	8 (3.8)
**Race**	
American Indian or Alaska Native	10 (4.8)
Asian	9 (4.3)
Black or African American	66 (31.6)
Native Hawaiian or other Pacific Islander	<5.0^†^
White	80 (38.3)
Multiracial or other	38 (18.2)
Unknown*	5 (2.4)
**Hispanic origin**	
Hispanic or Latino	59 (28.2)
Non-Hispanic	147 (70.3)
Unknown*	3 (1.4)
**Type of recruitment site**	
Encampment	16 (7.7)
Permanent supportive housing	30 (14.3)
Service center	68 (32.5)
Shelter	95 (45.5)

* Survey responses were indicated as unknown if the participant declined to answer the question or answered that they did not know.

^†^ The specific value for any cell with fewer than five participants is suppressed in accordance with data standards for the San Francisco Department of Public Health and are indicated within the cell as <5.0.

^§^ Among the 19 participants who identified as transgender women, 11 indicated that they were sexually oriented toward men (heterosexual), seven indicated that they were bisexual, gay or lesbian, or pansexual, and one declined to answer.

A total of 207 participants were included in serologic analysis, after exclusion of two participants (1%) who reported both previous mpox infection and mpox vaccination (Table 2). Among these participants, 82 (40%) reported previous vaccination against smallpox, mpox, or both, including 50 (24%) who reported only smallpox vaccination, 22 (11%) who reported only mpox vaccination, and 10 (5%) who reported both. Neither smallpox nor mpox vaccination was reported by 117 (60%) participants; vaccination status of eight (4%) was unknown. Among the 32 participants who reported any mpox vaccination, 26 (81%) reported receiving 1 dose, and six (19%) reported receiving 2 doses.

**TABLE 2:** Self-reported smallpox and mpox vaccination history and antiorthopoxvirus serology results among persons experiencing homelessness or accessing homeless services, who participated in an mpox seroprevalence survey — San Francisco, California, October–November 2022

Self-reported vaccination history*	No. (row %)			
	All participants	IgG-positive result^†,§^	Received IgM test^§^	IgM-positive result^†,¶^
**All participants**	**207 (100.0)**	**48 (23.2)**	**102 (48.8)**	**5 (4.9)**
Any smallpox or mpox vaccination	**82 (39.6)**	38 (46.3)	60 (73.2)	5 (8.3)
Only smallpox vaccination	**50 (23.9)**	19 (38.0)	31 (62.0)	1 (3.2)
Only mpox vaccination	**22 (11.5)**	12 (54.5)	19 (86.3)	4 (21.1)
Both smallpox and mpox vaccination	**10 (4.8)**	7 (70.0)	10 (100.0)	0 (—)
Unknown vaccination history	**8 (3.8)**	2 (25.0)	5 (62.5)	0 (—)
No smallpox or mpox vaccination	**117 (56.0)**	10 (8.5)	37 (31.6)	0 (—)
No mpox vaccination	**167 (80.7)**	27 (16.1)	68 (40.4)	1 (1.5)
Age >50 yrs, no smallpox vaccination	**37 (17.7)**	8 (21.6)	17 (45.9)	0 (—)
Age ≤50 yrs, no smallpox or mpox vaccination	**80 (38.6)**	2 (2.5)	24 (30.0)	0 (—)

**Abbreviations:** IgG = immunoglobulin G; IgM = immunoglobulin M.

* Categories are not mutually exclusive.

^†^ Optical density minus cutoff value ≥0.1.

^§^ Percentage of those with self-reported vaccination history.

^¶^ Percentage of participants who received antiorthopoxvirus IgM testing.

Among 50 participants who reported only smallpox vaccination, antiorthopoxvirus IgG was detected in 19 (38%) and IgM in one (3%). Among 22 participants who reported only mpox vaccination, antiorthopoxvirus IgG was detected in 12 (55%) and IgM in four (21%). Among 80 participants aged <50 years who did not report smallpox or mpox vaccination, two (3%) had detectable antiorthopoxvirus IgG. Among 68 participants without reported mpox vaccination who were tested for antiorthopoxvirus IgM, one (1.5%) had detectable antibodies. These results yielded a total of three possible undetected mpox infections.

None of these three participants reported sexual contact during the preceding month, being transgender, being a gay or bisexual man, or having a rash at the time of the survey (Table 3). Among the three participants with possible undetected mpox infection, two reported that during the previous month they had shared unwashed utensils, spent time around and touched someone with a rash, and shared smoking devices.

**TABLE 3:** Behaviors reported during the preceding month, by persons experiencing homelessness or accessing homeless services, who participated in an mpox seroprevalence survey* — San Francisco, California, October–November 2022

Behavior and frequency	No. (%)	
	All participants (N = 209)	Possible undetected mpox infection^†^ (n = 3)
**Shared unwashed cups, spoons, or forks**		
Never	**152 (72.7)**	1 (33.3)
A few times	**34 (16.3)**	2 (66.7)
Nearly every day	**8 (3.8)**	0 (—)
Every day	**15 (7.2)**	0 (—)
Unknown^§^	**0 (—)**	0 (—)
**Shared unwashed clothing or bedding**		
Never	**164 (78.5)**	3 (100.0)
A few times	**22 (10.5)**	0 (—)
Nearly every day	**7 (3.3)**	0 (—)
Every day	**16 (7.7)**	0 (—)
Unknown^§^	**0 (—)**	0 (—)
**Shared unwashed personal care items^¶^**		
Never	**185 (88.5)**	3 (100.0)
A few times	**15 (7.2)**	0 (—)
Nearly every day	**3 (1.4)**	0 (—)
Every day	**6 (2.9)**	0 (—)
Unknown^§^	**0 (—)**	0 (—)
**Spent time around someone with a rash, lesion, or sore**		
Never	**175 (83.7)**	1 (33.3)
A few times	**22 (10.5)**	2 (66.7)
Nearly every day	**5 (2.4)**	0 (—)
Every day	**7 (3.3)**	0 (—)
Unknown^§^	**0 (—)**	0 (—)
**Touched someone with a rash, lesion, or sore**		
Never	**195 (93.3)**	1 (33.3)
A few times	**10 (4.8)**	2 (66.7)
Nearly every day	**1 (<1.0)**	0 (—)
Every day	**2 (1.0)**	0 (—)
Unknown^§^	**1 (<1.0)**	0 (—)
**Had a rash, lesion, or sore at the time of survey**		
No	**181 (86.6)**	3 (100.0)
Yes	**28 (13.4)**	0 (—)
Unknown^§^	**0 (—)**	0 (—)
Injected drugs**		
Never	**160 (76.6)**	3 (100.0)
A few times	**16 (7.7)**	0 (—)
Nearly every day	**10 (4.8)**	0 (—)
Every day	**20 (9.6)**	0 (—)
Unknown^§^	**3 (1.4)**	0 (—)
**Shared smoking devices^††^**		
Never	**84 (40.2)**	1 (33.3)
A few times	**60 (28.7)**	1 (33.3)
Nearly every day	**16 (7.7)**	1 (33.3)
Every day	**47 (22.5)**	0 (—)
Unknown^§^	**2 (1.0)**	0 (—)
**Had vaginal, anal, or oral sex**		
No	**111 (53.1)**	3 (100.0)
Yes	**91 (43.5)**	0 (—)
Unknown^§^	**7 (3.3)**	0 (—)
**Engaged in sex work^§§^**		
No	**187 (89.5)**	3 (100.0)
Yes	**14 (6.7)**	0 (—)
Unknown^§^	**8 (3.8)**	0 (—)
**Where slept most often**		
Friend or family’s house	**9 (4.3)**	1 (33.3)
Hotel or motel	**2 (1.0)**	0 (—)
Private residence	**12 (5.7)**	0 (—)
Permanent supportive housing	**37 (17.7)**	0 (—)
Shelter or navigation center	**99 (47.4)**	2 (66.7)
Unsheltered	**46 (22.0)**	0 (—)
Other	**2 (1.0)**	0 (—)
Unknown^§^	**2 (1.0)**	0 (—)

**Abbreviations:** IgG = immunoglobulin G; IgM = immunoglobulin M.

* Most surveys (198; 94.7%) were conducted in English, 10 (4.8%) in Spanish, and one (0.5%) in Mandarin.

^†^ Possible undetected mpox infection was defined as detectable antiorthopoxvirus IgG antibody in a specimen from a person who did not report smallpox or mpox vaccination and was born after 1972 (aged <50 years), or in a specimen from a person with detectable antiorthopoxvirus IgM antibody who did not report mpox vaccination.

^§^ Survey responses are marked as unknown if the participant declined to answer the question or answered that they did not know.

^¶^ Based on response to the question, “In the last month have you shared unwashed personal care items (razors, toothbrushes, hairbrushes, etc.)?”

** Based on response to the question, “In the last month did you shoot up or inject any drugs - by shooting up, we mean using drugs with a needle, either by hitting a vein, skin popping, or muscling?”

^††^ Based on response to the question, “Have you shared bubbles/vapes/bongs/pipes/other smoking devices in the last month?”

^§§^ Based on responses to the following questions: 1) “Do you consider yourself to be a sex worker?” and 2) “In the last month did you have sex for money, drugs, food, housing, or other life needs?” Participants were classified as engaging in sex work if they responded affirmatively to either question.

## Discussion

Among 207 persons who were experiencing homelessness or accessing homeless services in San Francisco and voluntarily participated in an antiorthopoxvirus seroprevalence survey during a large multinational mpox outbreak, three possible undetected mpox infections were detected. Mpox infections might be undetected because of subclinical, atypical, or mild disease or because of barriers to seeking or accessing health care systems, which could have occurred among the participants in this survey. None of the participants with possible undetected mpox infections reported sexual contact during the preceding month, although some reported sharing utensils and smoking devices and spending time around or touching someone with a rash. However, the timing of mpox exposure among these three persons is not known and could have preceded the survey period. The transmission route for the three possible undetected mpox infections could not be determined; additional studies are needed to identify mpox transmission pathways among persons experiencing homelessness. In the current outbreak, mpox has primarily spread through sexual activity but can be spread through touching contaminated objects and through close personal contact outside of sexual activity, although this risk is considered to be lower (*6*).

Previous mpox vaccination was reported by 16% of survey participants. However, only 54% of participants reporting mpox vaccination had detectable antiorthopoxvirus IgG, and 21% had detectable IgM. A 2-dose mpox vaccination series is recommended to optimize immunity (*7*). Most participants who received mpox vaccine reported receiving a single dose, which might in part explain the lower seroprevalence (*2*,*7*). SFDPH and community partners collaborated to conduct pop-up vaccination events in San Francisco during September–December 2022 for persons experiencing homelessness; this active outreach approach can be further leveraged to better facilitate complete vaccination for eligible persons. A single mpox vaccine dose might still offer some protection against severe mpox-associated illness and hospitalization, for which persons experiencing homelessness (*1*,*7*,*8*) might be at higher risk.

The findings in this report are subject to at least seven limitations. First, participants were recruited as a convenience sample at prioritized locations; therefore, the findings are not generalizable to the entire population of persons experiencing homelessness or accessing homeless services in San Francisco. Second, the sample size was small because of time and resource limitations as well as logistical challenges associated with collecting blood samples from persons in encampments and at homeless service sites (*9*). Third, because of the small numbers of participants with potential undetected infection, statistical analyses could not be conducted to further refine possible transmission routes. Fourth, the survey ascertained frequency of behaviors during the preceding month to improve recall; however, it was not possible to determine whether behaviors that would increase mpox transmission risk were present outside that time frame. Fifth, the orthopoxvirus ELISA does not detect antibodies specific to *Monkeypox virus*; therefore, previous or acute mpox infection among the participants could not be distinguished from other orthopoxvirus infections or previous vaccination. Sixth, the survey relied on self-reported vaccination history and behaviors, which can be subject to recall and social desirability biases. Finally, it is possible for antibody testing to produce false-positive results. Efforts to improve specificity of possible mpox infection included restricting IgG results to persons aged <50 years to avoid inclusion of participants who received childhood smallpox vaccine, asking about previous military service to avoid inclusion of participants who received smallpox vaccination in the service, and using a higher OD-COV in ELISA tests to reduce risk for false-positive results (*5*,*10*). However, participants close to the cutoff age of 50 years could have received childhood smallpox vaccine, as was the case of one participant without reported vaccination history who had detectable antiorthopoxvirus IgG.

These findings suggest that undetected mpox infections might have occurred among a small percentage of persons experiencing homelessness in San Francisco. It is still unknown whether unique mpox transmission pathways exist for persons experiencing homelessness. However, given that known and possible undetected mpox transmission occurred, and that severe mpox disease among persons experiencing homelessness is possible (*1*), accessible prevention measures are needed. Prioritization and inclusion in public health response planning, focused outreach, and on-site vaccination events can ensure that prevention measures reach persons experiencing homelessness.

**Summary**

**What is already known about this topic?**

During the 2022 multinational outbreak of monkeypox (mpox), transmission occurred primarily through skin-to-skin contact, including through sex. Persons experiencing homelessness have been disproportionately affected by severe mpox.

**What is added by this report?**

Among 209 surveyed persons accessing homeless services or staying in encampments in San Francisco, California, without self-reported vaccination or reported previous mpox infection during October 25–November 3, 2022, three had detectable antiorthopoxvirus immunoglobulin (Ig) G or IgM, consistent with possible undetected mpox infection.

**What are the implications for public health practice?**

Possible undetected mpox among persons experiencing homelessness during the 2022 mpox outbreak highlights the need for tailored outreach strategies that ensure interventions such as prevention messaging and vaccination reach persons experiencing homelessness.

## Footnotes

* These authors contributed equally to this report.

† 45 C.F.R. part 46, 21 C.F.R. part 56; 42 U.S.C. Sect. 241(d); 5 U.S.C. Sect. 552a; 44 U.S.C. Sect. 3501 et seq.
